# Spontaneous Coronary Artery Dissection in the Setting of COVID-19 Pandemic-Related Stressors: A Case Report

**DOI:** 10.7759/cureus.23069

**Published:** 2022-03-11

**Authors:** Alexander M Roche, Kelsey Klingel, Kathryn Toth, Keating Pepper, Sanjeev A Francis

**Affiliations:** 1 Internal Medicine, Maine Medical Center, Tufts University School of Medicine, Boston, USA; 2 Internal Medicine, Maine Medical Center, University of New England College of Osteopathic Medicine, Biddeford, USA; 3 Emergency Medicine, Maine Medical Center, Portland, USA; 4 Anesthesiology, Maine Medical Center, Portland, USA; 5 Cardiology, Maine Medical Center, Portland, USA

**Keywords:** coronary fibromuscular dysplasia, acute st-elevation myocardial infarction, vaccine hesitancy, spontaneous coronary artery dissection, covid-19 vaccine, covid 19

## Abstract

Spontaneous coronary artery dissection (SCAD) is an uncommon but important cause of acute myocardial infarction, particularly in younger women and in patients with underlying fibromuscular dysplasia (FMD). There is increasing literature on patients with SCAD reporting significant emotional stress, particularly stress related to unemployment, in the week prior to their cardiac event, and emotional triggers appear to be associated with worse in-hospital and follow-up cardiac events. Additionally, the COVID-19 pandemic has resulted in significant societal stressors and increased unemployment, which have been associated with increased cardiovascular morbidity. Here, we present a case of a female presenting with an acute MI secondary to SCAD in the setting of recently learning of impending unemployment due to COVID-19 vaccine refusal. This case highlights the importance of considering SCAD in patients with significant recent emotional stress who present with MI. Additionally, in light of the emotional stressors of the COVID-19 pandemic, clinicians must be aware of the consequences significant emotional stress plays on the development of adverse complications of chronic disease.

## Introduction

Spontaneous coronary artery dissection (SCAD) is a rare but important cause of acute myocardial infarction and most often occurs as a complication of fibromuscular dysplasia (FMD) [1]. A large proportion of patients with SCAD report significant emotional stress immediately before the event and are more likely than patients with atherosclerosis-related ACS to report such stressors [2,3]. Furthermore, unemployment has been shown to be a significant stressor in patients with SCAD and is associated with worse in-hospital and follow-up cardiac events [4,5]. In the setting of increased stresses and unemployment during the COVID-19 pandemic, we present this case of a SCAD in a female with impending unemployment due to pandemic-related factors.

## Case presentation

A 54-year-old female with a past medical history of hypertension and hyperlipidemia presented to the emergency department with acute-onset substernal chest pain, pressure and nausea. On interview, both before treatment and after resolution of symptoms, she reported significant emotional stress, as she had recently learned that she and several family members were facing imminent unemployment, reportedly due to COVID-19 vaccine refusal.

**Figure 1:**
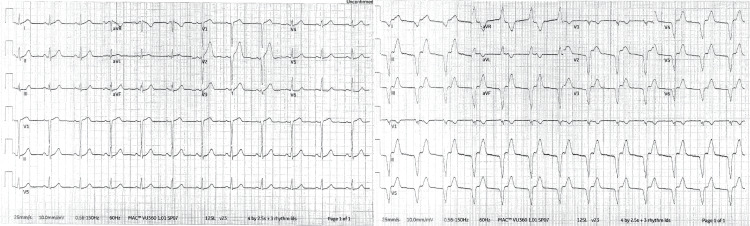
(Left) Initial EKG showing borderline ST elevations in anterior leads. (Right) Subsequent EKG showing accelerated interventricular rhythm.

Initial EKG showed borderline anterior ST-segment elevations and the team initiated nitroglycerin and heparin. Subsequent EKG showed accelerated idioventricular rhythm, potentially consistent with reperfusion (Figure 1).

Troponin I trended from undetectable on admission to 6.413 ng/mL (normal <0.04 ng/mL). Due to ongoing chest pain despite medical management, the patient was taken to the cardiac catheterization laboratory.

**Figure 2:**
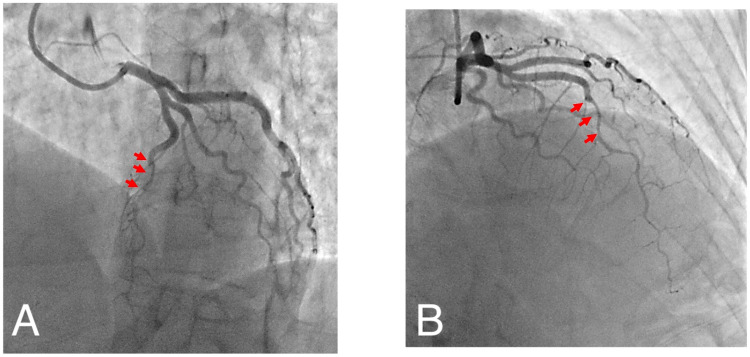
(A) Left anterior oblique cranial view of left coronary system. (B) Right anterior oblique cranial view of left coronary system. Note abrupt taper of mid LAD with small caliber vessel to the apex (arrows)

Coronary angiography demonstrated no coronary artery disease in the right coronary artery and left circumflex territory. The mid-lateral anterior descending artery showed a focal area of turbulent flow with a hazy appearance consistent with a dissection flap with evidence of an acute change in the caliber of the LAD from the mid-segment to the apical segment (Figures 2A, 2B), consistent with SCAD.

Left ventriculography demonstrated mildly reduced left ventricular systolic function with a large anteroapical akinetic segment. The patient was transferred to the CICU in guarded condition.

**Figure 3:**
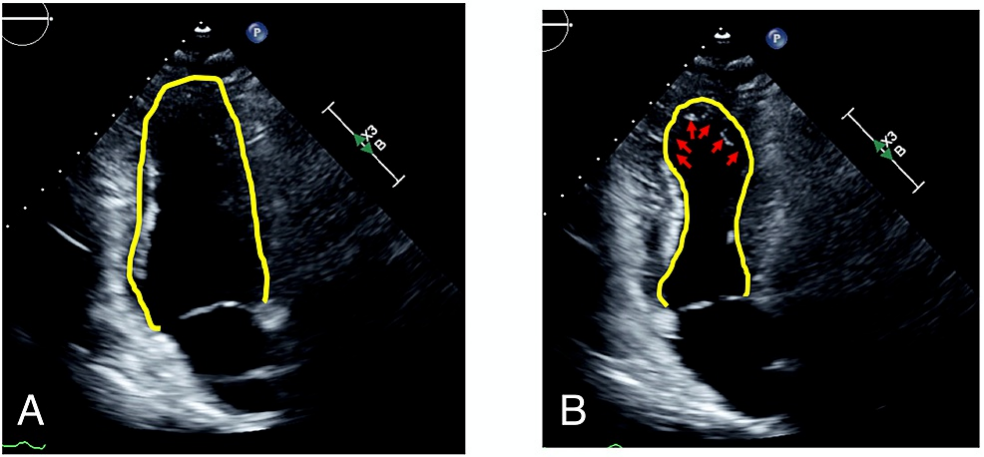
Transthoracic echocardiogram Apical 2 chamber view during diastole (A) and systole (B). Note apical anterior and apical inferior wall motion abnormalities.

In the CICU, dual-antiplatelet therapy and beta-blockade were started. Transthoracic echocardiogram revealed an estimated ejection fraction of 40%-45% with an anteroapical wall motion abnormality (Figures 3A, 3B).

**Figure 4:**
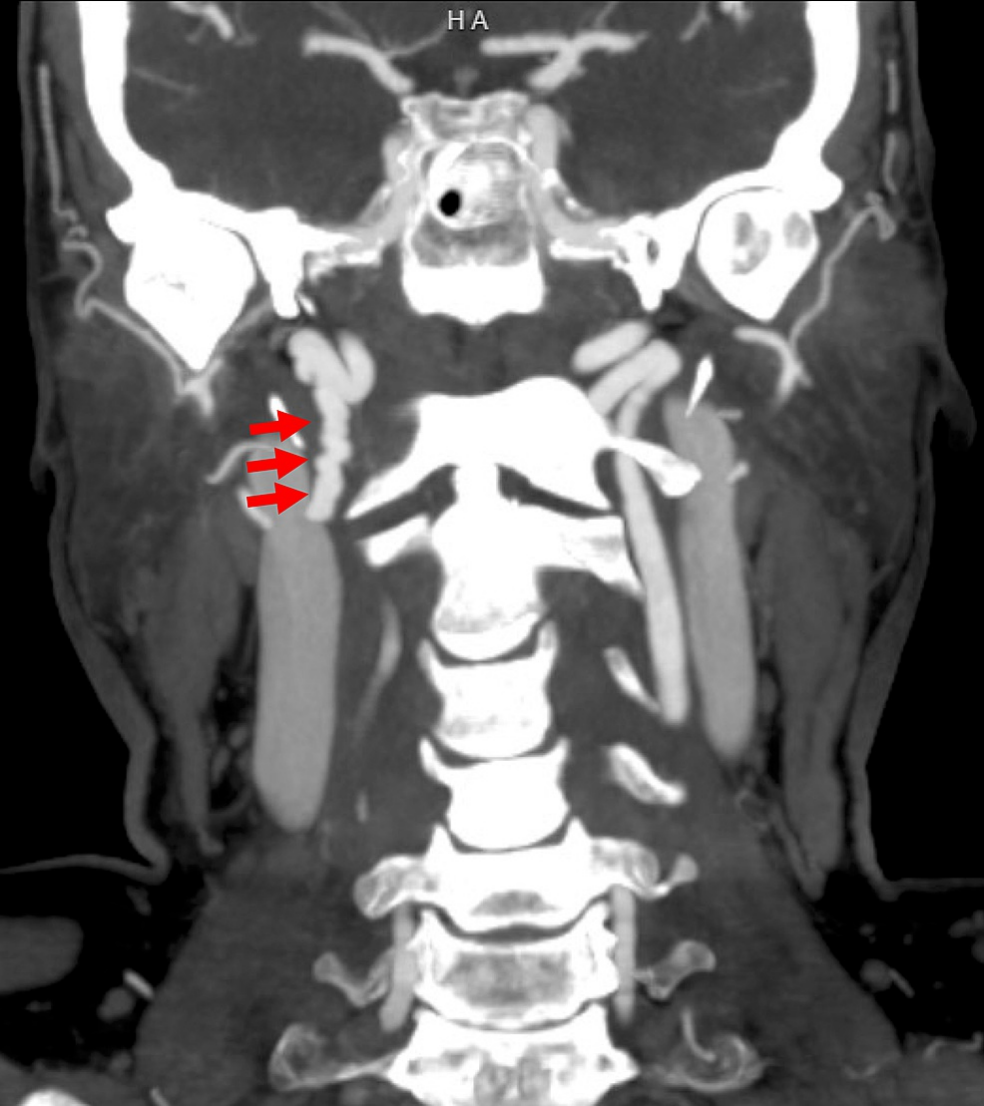
Neck CT angiogram shows coronal maximal intensity projection. Note the irregular beaded narrowing of the mid to distal cervical right ICA consistent with fibromuscular dysplasia.

The patient underwent a CT angiogram of the neck, chest, abdomen and pelvis to evaluate for FMD, which revealed irregular beading and significant stenosis of the right internal carotid artery, consistent with a diagnosis of FMD (Figure 4).

The patient remained stable without anginal symptoms through the remainder of her hospital course and was discharged on hospital day 3 with plans for follow-up.

## Discussion

The association between FMD and SCAD is well established, but SCAD is believed to be an uncommon initial presentation of FMD [6]. Therefore, recognition of risk factors, symptoms and findings is crucial to timely diagnosis and appropriate treatment of SCAD.

Myocardial infarction in a younger female should raise clinical suspicion for SCAD, as up to 30% of acute MI in women under the age of 50 have been found to be secondary to SCAD [7]. However, despite SCAD being thought of as a disease of younger, pre-menopausal women, post-menopausal women comprise a significant proportion of SCAD (up to 62.3% of cases) [3]. Post-menopausal women with SCAD were more likely to have underlying contributory health conditions such as hypertension and hyperlipidemia than pre-menopausal women but were equally likely to report emotional/physical triggers for SCAD and did not suffer from worse in-hospital or follow-up outcomes [8]. Additionally, patients of all ages with MI due to SCAD are more likely (56% vs. 39%) to report emotional stress immediately preceding their event than those with MI from atherosclerotic coronary artery disease, and a majority of women with SCAD reported significant emotional stress in the week leading up to their event [2]. Therefore, women presenting with MI in the setting of recent significant emotional stress should further raise clinical suspicion of SCAD. There is also some evidence that unemployment is a stressor significantly associated with SCAD, as patients with recent unemployment-related stress suffered worse in-hospital and follow-up cardiac events compared to those with other stressors [4,5].

In the patient presented here, the fact her SCAD coincided with COVID-19-related stressors, along with the substantial body of literature implicating emotional stress in the pathophysiology of SCAD, suggested that her stress may have contributed to her disease process, along with underlying risk factors such as hypertension and hyperlipidemia. As such, treatment of her condition requires ongoing psychosocial support, including education about her condition, in addition to traditional medical interventions. The American Heart Association (AHA) emphasizes the importance of addressing mental health in the wake of a SCAD, as patients who suffer from SCAD have high rates of anxiety and depression as a result of their diagnosis [9]. Factors that may contribute to these high rates include prognostic uncertainty, risk of recurrence, lack of understanding from doctors/peers due to the rarity of the disease and a lack of understanding of the disease by the patient. Therefore, the AHA recommends ongoing psychosocial and community support to address underlying causes of anxiety in patients recovering from SCAD.

The COVID-19 pandemic has led to significant societal stressors and deteriorating global mental health, with new diagnoses of major depressive disorder and generalized anxiety disorder increasing by an estimated 25%-28% in 2020 [10]. Additionally, the onset of the pandemic caused a significant increase in unemployment in the United States; the nationwide unemployment rate in April 2020 was 14.8% [11], the highest level since the 1930s [12], and unemployment in September 2021 (4.8%, but thought to be an underestimation) is still higher than pre-pandemic levels in February 2020 (3.5%) [12]. Pandemic-related stressors, including those related to unemployment, may play an increasingly large role in overall population stress during the pandemic and may contribute to certain medical pathologies with stress-induced etiologies. Of note, there have been multiple reports of cardiac events secondary to pandemic-related stressors, including increased cases/rates of Takotsubo cardiomyopathy [13,14], and established risk factors for cardiovascular disease have increased over the course of the pandemic [15]. Though the mechanism by which severe emotional stress incites cardiac diseases including Takotsubo cardiomyopathy and SCAD has not been fully investigated, stress-induced catecholamine surges leading to coronary artery shear stress are thought to contribute significantly to the underlying pathophysiology [9]. Therefore, ongoing management of emotional stressors, especially given the prevalence of such stressors in the pandemic, may be crucial in the proper treatment of these conditions.

## Conclusions

Emotional stressors related to the COVID-19 pandemic may result in morbidity and mortality beyond the immediate effects of the pandemic. As illustrated by the case presented here, pathologies such as SCAD that are often induced by severe emotional stress may present more frequently during the pandemic and may often be associated with pandemic-related stressors, including unemployment. Therefore, health providers caring for patients during the pandemic should note that the distinct, difficult circumstances of the pandemic may play a significant role in inciting or exacerbating disease and should learn to recognize and inquire about such stressors when working up acute medical conditions. Furthermore, short- and long-term management of these patients should include psychosocial interventions to address the underlying causes of their emotional stressors in addition to usual medical management, particularly in conditions such as SCAD that hold a high risk of recurrence with ongoing stressors.

## References

[1] Saw J, Mancini GB, Humphries KH (2016). Contemporary review on spontaneous coronary artery dissection. J Am Coll Cardiol.

[2] Smaardijk VR, Mommersteeg PM, Kop WJ, Pellegrini D, van Geuns RJ, Maas AH (2021). Psychological and clinical characteristics of patients with spontaneous coronary artery dissection: a case-control study. Int J Cardiol.

[3] Saw J, Aymong E, Sedlak T (2014). Spontaneous coronary artery dissection: association with predisposing arteriopathies and precipitating stressors and cardiovascular outcomes. Circ Cardiovasc Interv.

[4] Alipour S, Starovoytov A, Heydari-Kamjani M (2016). Frequency and effects of emotional and physical stressors in patients with spontaneous coronary artery dissection. J Am College Cardiol.

[5] Daoulah A, Al-Faifi SM, Hurley WT (2021). Spontaneous coronary artery dissection: does being unemployed matter? Insights from the GSCAD Registry. Curr Cardiol Rev.

[6] Farooq A, Amjad W, Bajwa AU, Yasin H, Ali R, Pervaiz M (2017). Fibromuscular dysplasia with spontaneous coronary artery disease presenting as acute myocardial infarction. Cureus.

[7] Saw J, Aymong E, Mancini GB, Sedlak T, Starovoytov A, Ricci D (2014). Nonatherosclerotic coronary artery disease in young women. Can J Cardiol.

[8] Díez-Villanueva P, García-Guimaraes MM, Macaya F (2021). Spontaneous coronary artery dissection and mnopause. Am J Cardiol.

[9] Hayes SN, Kim ES, Saw J (2018). Spontaneous coronary artery dissection: current state of the science: a scientific statement from the American Heart Association. Circulation.

[10] Collaborators CMD (2021). Global prevalence and burden of depressive and anxiety disorders in 204 countries and territories in 2020 due to the COVID-19 pandemic. Lancet.

[11] (2021). Civilian unemployment rate. https://www.bls.gov/charts/employment-situation/civilian-unemployment-rate.htm2021..

[12] (2021). Tracking the COVID-19 economy’s effects on food, housing, and employment hardships. https://www.cbpp.org/research/poverty-and-inequality/tracking-the-covid-19-economys-effects-on-food-housing-and2021..

[13] Jabri A, Kalra A, Kumar A (2020). Incidence of stress cardiomyopathy during the coronavirus disease 2019 pandemic. JAMA Netw Open.

[14] Mohammed M, Zakhour S, Devgun J, Lee J, Keimig T, Wang DD (2020). Takotsubo cardiomyopathy in a healthcare worker during the COVID-19 pandemic: caused by the virus or the demands of the many being placed on the few?. Eur J Case Rep Intern Med.

[15] Mattioli AV, Nasi M, Cocchi C, Farinetti A (2020). COVID-19 outbreak: impact of the quarantine-induced stress on cardiovascular disease risk burden. Future Cardiol.

